# Prevalence of gastric cancer following colorectal endoscopic submucosal dissection for lesions more than 20 mm: A retrospective analysis

**DOI:** 10.1002/deo2.70042

**Published:** 2024-12-18

**Authors:** Yuri Tomita, Naohisa Yoshida, Hideki Ishikawa, Takahiro Otani, Reo Kobayashi, Hikaru Hashimoto, Ryohei Hirose, Osamu Dohi, Ken Inoue, Yukiko Morinaga, Yoshito Itoh

**Affiliations:** ^1^ Department of Gastroenterology Koseikai Takeda Hospital Kyoto Japan; ^2^ Department of Molecular Gastroenterology and Hepatology Kyoto Prefectural University of Medicine Graduate School of Medical Science Kyoto Japan; ^3^ Department of Molecular‐Targeting Prevention Kyoto Prefectural University of Medicine Graduate School of Medical Science Kyoto Japan; ^4^ Department of Public Health Nagoya City University Graduate School of Medical Sciences Aichi Japan; ^5^ Department of Gastroenterology Osaka General Hospital of West Japan Railway Company Osaka Japan; ^6^ Department of Surgical Pathology Kyoto Prefectural University of Medicine Graduate School of Medical Science Kyoto Japan

**Keywords:** colorectal neoplasms, colonoscopy, endoscopic submucosal dissection, esophagogastroduodenoscopy, gastric cancer

## Abstract

**Objectives:**

Colorectal endoscopic submucosal dissection (ESD) for large tumors is spreading worldwide. Gastric cancer (GC) sometimes occurs after colorectal ESD. However, its status including frequency and risk factors have not been examined well. In this study, we analyzed the detailed status of GC after colorectal ESD.

**Methods:**

This was a single‐center retrospective study. Patients receiving colorectal ESD between 2010 and 2018 were reviewed. All patients were recommended to receive esophagogastroduodenoscopy (EGD) for screening. Finally, 436 patients receiving EGD, who underwent colorectal ESD for lesions of ≥20 mm were analyzed. The primary outcome was the GC rate after colorectal ESD, including intramucosal cancer. As a control, we compared it to the GC rate in matched Japanese national cancer registry data. The secondary outcome was risk factors for developing GC.

**Results:**

The mean age was 66.9 ± 10.6 and 55.3% were males. The GC rate was 5.96% (26/436) with a median observation period of 27 months. It was significantly higher than the mean GC rate in the diagnosed age calculated with the cancer registry (0.26%, observed value/expected value ratio [95% confidence interval]: 22.20 [14.50–32.53], *p* < 0.01). The comparison between cases with and without GC showed that significant risk factors were male (*p* = 0.02) and smokers (*p* < 0.01) and their GC rates were 8.3% and 10.9%. Also, in the limited cases, *Helicobacter pylori* infection (past and present) and atrophic gastritis were significant and their GC rates were 11.1% and 11.6%.

**Conclusion:**

The GC rate was high after resecting colorectal tumors of ≥20 mm, suggesting the necessity of EGD.

## INTRODUCTION

Globally, in terms of cancer incidence in 2022, colorectal cancer (CRC) ranked third with 1,926,425 cases and gastric cancer (GC) ranked fifth with 968,784 cases.^1^ The numbers of CRC and GC in 2018 were 1,096,601 and 1,033,701. In 2018, the incidence of CRC in Japan was 155,625 people, the highest among all cancers, and the number of GC was 124,319 people, which was the third highest.^2^ The number of CRC and GC in 2012 was 134,575 and 132,159, respectively. Thus, the number of CRCs is increasing and that of GC is decreasing in the world and Japan.

Endoscopic submucosal dissection (ESD) is increasing worldwide enabling en‐bloc resection of large colorectal tumors.^3^, ^4^ GC is sometimes discovered during the surveillance period after colorectal ESD. However, various guidelines do not recommend esophagogastroduodenoscopy (EGD) for screening when treating CRC either with endoscopy or surgery.^5^, ^6^, ^7^, ^8^ EGD is performed as cancer screening in Japan and the GC rate by EGD as cancer screening for general people was reported 0.91% (143/15763).^9^ Old age, smoking, and *Helicobacter pylori* *(H. Pylori)* infection have been identified as risk factors for GC.^10^, ^11^, ^12^ Processed meat, excessive alcohol consumption, obesity, excessive drinking, lack of exercise, and smoking are recognized as risk factors for CRC.^13^, ^14^ Thus, smoking is overlapped as a risk factor for both cancers. However, to date, the existence of CRC and large colorectal tumors has not been reported to be risk factors for GC. We previously reported a significantly higher rate of metachronous GC in patients receiving endoscopic resection for colorectal lesions ≥20 mm compared to those for lesions <20 mm using large‐scale health insurance claims data in Japan.^15^ In this study, we analyzed the detailed status of GC after real colorectal ESDs.

## METHODS

**FIGURE 1:**
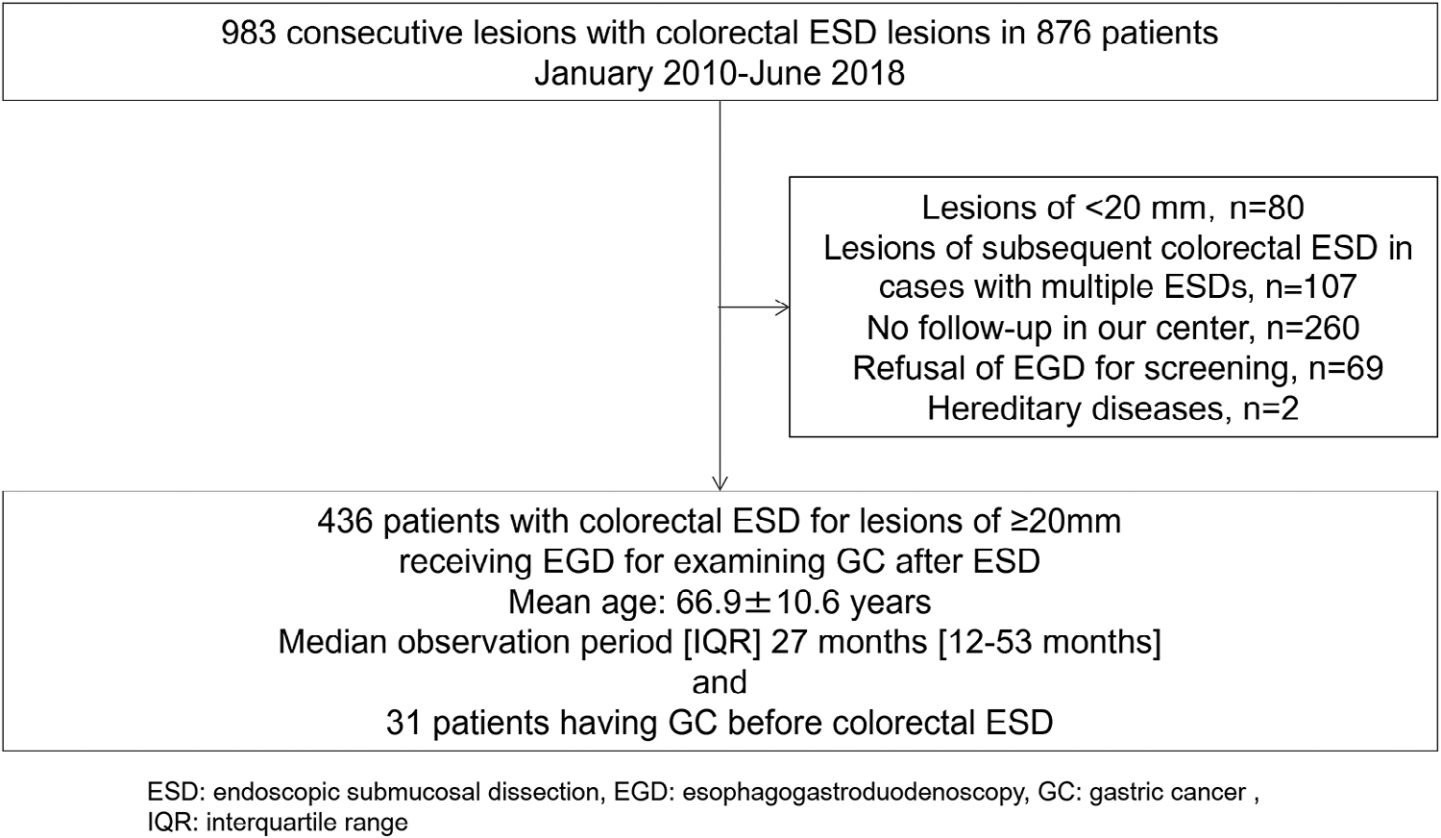
The flow diagram of the present study.

This was a retrospective single‐center study. We examined 983 lesions in 876 patients who underwent colorectal ESD at our hospital between January 2010 and June 2018 (Figure 1). In this period, EGD was recommended for all patients for screening after colorectal ESD. A first EGD was regularly recommended within 1 year after ESD and then, EGD was asked every 1–2 years according to risk factors of GC.^10^, ^11^, ^12^ Based on the Japanese guidelines, the indications for colorectal ESD were (1) lesions ≥20 mm diagnosed endoscopically as Tis or T1a, or (2) lesions <20 mm judged to be difficult to resect by EMR owing to severe fibrosis or submucosal invasion.^3^ The rate of GC was examined until February 2022. Regarding the case registration period, we examined cases receiving ESD from 2010 to 2018 because the data before 2009 did not include enough information. Exclusion criteria included colorectal tumors <20 mm (80 cases), lesions of subsequent colorectal ESD in cases with multiple ESDs (107 cases), no follow‐up in our center (260 cases), refusal of EGD for screening (69 cases) and hereditary diseases (two cases). Finally, we analyzed 436 cases who received EGD for examining GC after colorectal ESD after exclusion (Figure 1).

### Endpoints

The primary endpoint was the rate of GC after colorectal ESD compared with the rate in the general Japanese population calculated with the cancer registry. Secondary endpoints were as follows: (1) the analysis of risk factors such as age, sex, life habits, body mass index, serum carcinoembryonic antigen (CEA), diabetes, and history of malignant disease, colorectal tumors characteristics, GC lesions characteristics and *H. pylori* infection (past and present), and atrophic gastritis, for developing GC after colorectal ESD comparing the GC and non‐GC cases, (2) the cumulative rate of GC for overall patients and the rates of patients with risk factors discovered for the development of GC, (3) the analysis of GC cases before and after colorectal ESD, including location, size, histopathological subtype, tumor depth, the existence of atrophic gastritis, and the time from ESD to detecting GC.

The rate of GC in the Japanese population was estimated according to data from the National Cancer Registry of Japan in 2018.^2^ The data provided the incidence of GC by person's age category (every 5 years) and sex every year at diagnosis (2016–2019). We only matched our patient's sex, age, and EGD date to the year of diagnosis to the National Cancer Registry data though we did not extract individual cases from it by matching sex, age, and EGD year with each subject. The national data only provided GC diagnosis frequencies from 2016 to 2019. Thus, for patients who underwent EGD before 2015, we matched cases to 2016 data, and for those after 2020, to 2019 data. We adopted the last EGD for patients who received multiple EGD. For example, a male case receiving colorectal ESD in 2014 and receiving the last EGD after ESD in 2020 (his age was 64 years old at the EGD) was matched to a male case aged 60–64 years category in the 2019 data. Then, the GC rate of this case was estimated according to the matched case. Accordingly, we calculated the estimated mean rate of GC in the year of diagnosis for those people after matching and comparing the data to the real rate obtained from our patients. We also calculated the estimated mean cumulative rate of GC for those people, summing all GC incidences by age category (every 5 years) from 0 years old to EGD date and compared the real rate. In this analysis, we calculated the real number of GC (observed value)/expected number of GC described above (expected value) as an O/E ratio.

Present smokers and ex‐smokers with a Brinkman index ≥400 were defined as smokers according to a paper.^16^ People drinking pure alcohol amount >120 g/ week are defined as drinkers, modifying the World Health Organization (WHO) statement because Japanese people frequently had weak effects of alcohol metabolizing enzymes.^17^, ^18^ The colorectal tumor location was divided into three types: right‐side colon (cecum to transverse colon), left‐side colon (descending colon to sigmoid colon), and rectum. The morphology type was classified into polypoid and nonpolypoid types based on the Paris classification.^19^ Colorectal lesion size was calculated according to resected specimens with ESD. The *H. pylori* infection (past and present) was detected by the urea breath test, serum *H. pylori* antibody test, or the patient's history of *H. pylori* eradication from the electrical record system. Atrophic gastritis was diagnosed endoscopically according to the Kimura‐Takemoto classification by recorded endoscopic images and atrophic grade was classified into close type and open type.^20^ The tumor location of GC was divided into three parts: the upper part (cardia and fundus), the middle part (corpus), and the lower part (antrum and pylorus).

Histopathological diagnosis for colorectal lesions and GC was performed according to the WHO classification and the Japanese guidelines.^21^, ^22^, ^23^ The type of GC was classified as well (well‐moderately differentiated tubular and papillary adenocarcinoma), poor (poorly differentiated carcinoma and signet‐ring cell carcinoma).

This study was conducted following the World Medical Association Declaration of Helsinki and was approved by the institutional review board and ethics committees of Kyoto Prefectural University of Medicine (ERB‐C‐1944). This study was retrospective, and the opt‐out approach was adopted, using their website or a notice in the posting area.

### Statistical analyses

The Mann–Whitney U test or chi‐squared test was used for statistical analysis. After matching, the expected numbers of GC cases in the population were calculated based on the mean rate and the mean cumulative rate to obtain O/E ratios, and 95% confidence intervals (95% CIs) and *p*‐values for the ratios were obtained using Byar's method. The Kaplan–Meier analysis of the cumulative rate of GC was performed and the comparison of sex and smoking was performed using the log‐rank test. Multivariate analysis was performed using the Cox proportional hazards model and age, sex, and variables with *p* < 0.05 in the univariate analysis were included. Prior to conducting the multivariate analysis, variables exhibiting high multicollinearity were excluded. Statistical significance was set at *p* < 0.05. All statistical analyses were performed using statistical software (SPSS software, version 22.0; IBM Japan Ltd. or R version 4.2.2 for Windows; R Foundation for Statistical Computing)

## RESULTS

Among 436 patients, the mean age was 66.9 ± 10.6 years and the male ratio was 55.3% (241/436; Table 1). Histopathological diagnosis of Tis+T1≤ in colorectal ESD was 60.6% (264/436).

**TABLE 1:** Patient characteristics of all 436 cases.

Patient number	436
Lesion number	436
Age (years), mean ± SD (range)	66.9 ± 10.6 (22–90)
<75 years old/≥75 years old, % (*n*)	74.8/25.2 (326/110)
Sex, male/female, % (*n*)	55.3/44.7 (241/195)
Smoker, % (*n*)	27.3 (119/436)
Drinker, % (*n*)	32.6 (142/436)
BMI, mean ± SD	22.5 ± 3.5
Diabetes mellitus, % (*n*)	14.7 (64/436)
Past history of malignant disease, % (*n*)	20.9 (91/436)
Colorectal tumor location, right‐side/left‐side/rectum, % (*n*)	49.8/20.0/30.3 (217/87/132)
Colorectal tumor morphology, polypoid/nonpolypoid, % (*n*)	20.9/79.1 (91/345)
Colorectal tumor size, mm, mean ± SD (range)	32.7 ± 35.9 (20–140)
Colorectal tumor histopathology, SSL/adenoma/Tis/T1, % (*n*)	4.9/34.6/45.0/15.6 (21/151/196/68)
*H. pylori* infection, negative/positive/unknown, *n*	75/180/181
*H. pylor* *i* infection (past and present) in the identified case, % (*n*)	70.6 (180/255)
Atrophic gastritis, negative‐positive/unknown	75/173/188
Atrophic gastritis, % (*n*)	69.8 (173/248)
Atrophic grade, close type/open type, %, (*n*)	45.7/54.3 (79/94)
Median observation period, month [IQR]	27 [12–53]

Abbreviations: BMI, body mass index; *H. pylori*, *Helicobacter pylori*; IQR, interquartile; left‐sided, descending colon to sigmoid colon; right‐sided, cecum to transverse colon; SD, standard deviation; SSL, sessile serrated lesion.

The rate of GC after colorectal ESD was 5.96% (26/436) with a median observation period [interquartile range] of 27 months [12–53 months] (Table 2). The rate was significantly higher than both the mean rate of GC in the diagnosed age group (0.26%, O/E ratio (95% CI): 22.20 [14.50–32.53] (*p* < 0.01) and the estimated mean cumulative rate of GC (3.37%, O/E ratio (95% CI): 1.76 [1.15–2.58], *p* < 0.01) in the Japanese population.

**TABLE 2:** The rate of gastric cancer after resecting lesions ≥20 mm with colorectal endoscopic submucosal dissection compared to the expected rate in the regular Japanese population using the National Cancer Registry.

Number (O)/ rate of GC	Expected number of GC (E)	O/E ratio	95% CI, *p*‐value	After matching
26/ 5.96%	1.17	22.20	14.50–32.53, <0.01	Estimated mean rate of GC in diagnosed age after was 0.26%
	14.72	1.76	1.15–2.58, <0.01	Estimated mean cumulative rate of GC was 3.37%

Abbreviations: E, expected value; ESD, endoscopic submucosal dissection; GC, gastric cancer; O, observed value; 95% CI, 95% confidence interval.

Regarding comparing GC and non‐GC groups, the male ratios were 76.9% and 53.9% (*p* = 0.02; Table 3 and Supporting Information Figure). The rates of smokers were 53.8% and 29.8% (*p* = 0.01). *H. pylori* infection (past and present) in the 255 identified cases and atrophic gastritis in the 248 identified cases were also significant (*p* = 0.02 and *p* = 0.04) and the GC rates for them were 11.1% and 11.6%. In a multivariate analysis, only *H. pylori* infection was significant (hazard ratio: 5.05: 95% CI: 1.97–13.00, *p* < 0.01).

**TABLE 3:** The comparison of clinical characteristics of cases with gastric cancer and without gastric cancer after colorectal endoscopic submucosal dissection.

	Univariate analysis				Multivariate analysis		
	GC	Non‐GC	Rate of GC, % (*n*)	*p*‐value	Hazard ratio	95% CI	*p*‐value
Overall	26	410	5.9 (26/436)				
Age, mean ± SD (range)	67.1 ± 13.5 (22‐82)	66.9 ± 10.3 (35‐90)		0.45			
<75 years old, % (*n*)	69.2 (18)	75.1 (308)	5.5 (18/326)	0.50			
≥75 years old, % (*n*)	30.8 (8)	24.9 (102)	7.3 (8/110)				
Sex							
Male, % (*n*)	76.9 (20)	53.9 (221)	8.3 (20/241)	0.02	1.98	0.72–5.43	0.18
Female, % (*n*)	23.1 (6)	46.1 (189)	3.1 (6/195)				
Smoker, % (*n*)	53.8 (14)	29.8 (122)	10.3 (14/136)	0.01	2.28	0.95–5.46	0.06
Non‐smoker, % (*n*)	46.2 (12)	70.2 (288)	4.0 (12/300)				
Drinker, % (*n*)	46.2 (12)	31.7 (130)	8.5 (12/142)	0.12			
Non‐drinker, % (*n*)	53.8 (14)	60.3 (280)	4.8 (14/294)				
BMI, mean ± SD	22.7 ± 3.5	22.5 ± 3.4		0.91			
Diabetes mellitus, % (*n*)	19.2 (5)	14.4 (59)	7.8 (5/64)	0.49			
Past history of malignant disease, % (*n*)	26.9 (7)	20.5 (84)	7.7 (11/91)	0.43			
Colorectal tumor location							
Right‐side, % (*n*)	46.2 (12)	50.0 (205)	5.5 (12/217)	0.90			
Left‐side, % (*n*)	23.0 (6)	19.8 (81)	6.9 (6/87)				
Rectum	30.8 (8)	30.2 (124)	6.1 (8/132)				
Colorectal tumor morphology							
Polypoid, % (*n*)	19.2 (5)	21.0 (86)	5.5 (5/91)	0.83			
Nonpolypoid, % (*n*)	80.8 (21)	79.0 (324)	6.1 (21/345)				
Colorectal tumor size, mm, mean ± SD	33.5 ± 12.6	32.6 ± 15.0		0.36			
Colorectal histopathology							
SSL+adenoma, % (*n*)	26.9 (7)	40.2 (165)	4.1 (7/172)	0.17			
Tis+T1, % (*n*)	73.1 (19)	59.8 (245)	7.2 (19/264)				
T1, % (*n*)	23.1 (6)	15.1 (62)	8.8 (6/68)	0.27			
*H. pylori* infection (past and present), % (*n*)	90.9 (20)	68.7 (160)	11.1 (20/180)	0.02	5.05	1.97–13.00	<0.01
Atrophic gastritis, % (*n*)	90.9 (20)	67.7 (153)	11.6 (20/173)	0.04	n.c.		
Median observation period, month [IQR]	11.5 [2‐45.5]	12.5 [12‐47]		0.83			

Abbreviations: BMI, body mass index; CI, confidence interval; ESD, endoscopic submucosal dissection; GC, gastric cancer; IQR, interquartile; left‐sided, descending colon to sigmoid colon; n.c., not calculated for multicollinearity; right‐sided, cecum to transverse colon; SD, standard deviation; SSL, sessile serrated lesion.

**FIGURE 2:**
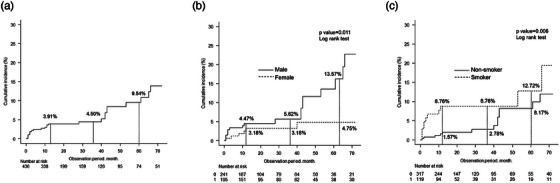
The cumulative rate of gastric cancer (GC) about sex and smoking. (a) The overall rate; (b) The rates about sex; (c) The rates about smoking.

The overall cumulative rate was 3.91%, 4.50%, and 9.54% one year, three years, and five years after colorectal ESD (Figure 2a). There were significant differences in the rate of sex (*p* = 0.01) and smoking (*p* < 0.01; Figure 2b,c).

The atrophic grade of cases with GC and without GC after colorectal ESD was examined (Table 4). There was a significant difference in the rate of open type between the GC and non‐GC groups (*p* < 0.01). The rate of GC was 17.0% in open‐type atrophic gastritis.

**TABLE 4:** The atrophic grade of cases with gastric cancer and without gastrid cancer after colorectal endoscopic submucosal dissection.

	GC	Non‐GC	Rate of GC, % (*n*)	*p*‐value
Atrophic grade close type, % (*n*)	18.2 (4/22)	33.2 (75/226)	5.1 (4/79)	0.22
Atrophic grade open type, % (*n*)	72.7 (16/22)	34.5 (78/226)	17.0 (16/94)	<0.01

Abbreviations: ESD, endoscopic submucosal dissection; GC, gastric cancer.

We compared 31 cases of GC before colorectal ESD with 26 cases of GC after colorectal ESD (Table 5). The mean age was 74.4 ± 7.9 vs. 67.1 ± 13.5 (*p* = 0.01). The mean body mass index was 20.7 ± 2.7 vs. 22.7 ± 3.5 (*p* < 0.01), respectively. The mean tumor size of GC (*p* = 0.03) and the tumor depth of GC (*p* < 0.01) were significant.

**TABLE 5:** The comparison of clinical characteristics of cases with gastric cancer before and after colorectal endoscopic submucosal dissection.

	Cases of GC before colorectal ESD (*n* = 31)	Cases of GC after colorectal ESD (*n* = 26)	*p*‐value
Patient number	34, 6.6% (31/467)	26, 6.0% (26/467)	0.49
Age (years), mean ± SD (range)	74.4 ± 7.9 (40‐86)	67.1 ± 13.5 (22–82)	0.01
<75 years old/≥75 years old, % (n)	38.7/61.3 (12/19)	69.2/30.8 (18/8)	0.02
Sex, male/female, % (*n*)	71.0/29.0 (22/9)	76.9/23.1 (20/6)	0.83
Smoker, % (*n*)	38.7 (12)	65.4 (13)	0.39
Drinker, % (*n*)	32.3 (10)	46.2 (12)	0.28
BMI, mean ± SD (range)	20.7 ± 2.7 (15.3–32.0)	22.7 ± 3.5 (16.2–32.0)	<0.01
CEA, mean ± SD (range), ng/ml	6.5 ± 14.8 (0.8–73.1)	2.8 ± 2.1 (0.9–12.1)	0.08
Colorectal tumor location, right‐side/left‐side/rectum, % (*n*)	61.3/22.6/16.1 (19/7/5)	46.2/23.1/30.8 (12/6/8)	0.38
Colorectal tumor morphology, polypoid/nonpolypoid, % (*n*)	9.7/90.3 (3/28)	19.2/80.8 (5/21)	0.51
Colorectal tumor size, mm, mean ± SD (range)	33.5 ± 17.3 (20–85)	33.5 ± 12.6 (20–80)	0.49
Colorectal histopathology, SSL+adenoma/Tis/T1, % (*n*)	45.2/45.2/9.7 (14/14/3)	26.9/50.0/23.1 (7/13/6)	0.58
GC Lesion number, *n*	33	32	
Tumor location, U/M/L/Unknown case, % (*n*)	7.7/38.5/53.8 (2/10/14/7)	13.8/62.1/24.1 (4/18/7/3)	0.08
Tumor size, mm, mean ± SD	29.1 ± 18.3	19.0 ± 16.5	0.03
Histopathological subtype, well/poor/unknown, % (*n*)	51.5/9.1/39.4 (17/3/13)	71.9/18.8/9.4 (23/6/3)	<0.01
Tumor depth, Tis/T1 or deeper, % (n)	36.4/63.6 (12/21)	71.9/28.1 (23/9)	<0.01
Atrophic gastritis, % (*n*)	*93.7 (15/16)	*90.9 (20/22)	0.93
Time from ESD to detecting GC, month, mean ± SD,	71.6 ± 87.5	27.9 ± 29.0	<0.01
<3 years/≥3 years, % (*n*)	54.8/45.2 (17/14)	57.7/42.3 (15/11)	0.82

Abbreviations: BMI, body mass index; CEA, carcinoembryonic antigen; ESD, endoscopic submucosal dissection, GC, gastric cancer; L, lower part; LDL‐C, low‐density lipoprotein cholesterol; left‐sided, descending colon to sigmoid colon; M, middle part; poor, signet‐ring cell carcinoma or poorly differentiated carcinoma; right‐sided, cecum to transverse colon; SD, standard deviation; SSL, sessile serrated lesion; TG, triglyceride; U, upper part; well, well‐moderately tubular or papillary adenocarcinoma.

## DISCUSSION

GC was reported as the most frequent cancer (1%–3%) after resection of CRC in Japan, Korea, and Germany and it was more common in men.^24^, ^25^, ^26^ In another study, the incidence of GC was significantly higher in patients who underwent surgery for CRC (follow‐up period: 28.3 ± 25.11 months, GC: 2.3%) than in the control group without CRC (follow‐up periods: 44.4 ± 25.37 months, GC: 0.5%, *p* = 0.002).^27^ We also reported the rate of GC after endoscopic resection for colorectal lesions using large‐scale Japanese health insurance claims data including 5,710,000 patients data. The rate for colorectal lesions ≥20 mm (1.17%, median follow‐up: 23 months) was significantly higher than those for lesions <20 mm (0.65%, median follow‐up: 22 months).^15^ However, compared to these rates, the real incidence of GC in the Japanese general population was reported 0.91% by EGD and we hypothesized that there was a discrepancy between data using health insurance claims and real data.^9^ In the current study of real cases, the rate of GC after resection of colorectal lesions of ≥20 mm was substantially high (5.96%), comparing the estimated rate of diagnosed year (0.26%) and the cumulative rate (3.37%) from Japanese National Cancer Registry. This suggested colorectal lesions ≥20 mm are related to GC similar to our previous study.^15^ Based on our findings, patients undergoing treatment for large colorectal lesions should be evaluated with EGD to monitor for the development of GC.

We emphasized the size of resected colorectal lesions instead of histopathology as the gold standard for various evaluations. Because the histopathological diagnosis is sometimes subjective and different for each pathologist even if WHO classification is adopted. Oppositely, we considered that lesion size is more objective than histopathological diagnosis. For lesion size, all resected specimens of ESD were pinned in our institution and calculated accurately. Regarding the analysis according to histopathological diagnosis, the rates of GC of CRC (Tis+ ≤T1) and benign tumors (SSL+adenoma) were not significant (7.2% vs. 4.1%, *p* = 0.17). Thus, the histopathological diagnosis of CRC was not related to metachronous GC. After resecting colorectal lesions ≥20 mm, we also reported that metachronous advanced adenoma occurred significantly more than lesions <20 mm.^28^ Thus, patients with colorectal lesions measuring ≥20 mm may have malignant potential affecting various organs. Further large‐scale research, free from selection bias, is necessary to substantiate this finding.

Besides colorectal lesions ≥20 mm, age ≥65 years, male gender, diabetes mellitus, liver disease, and *H. pylori* infection were also significant risk factors for GC after colonoscopic resection in our previous study.^29^ Older age and male gender have been well reported as risks for GC.^10^, ^12^, ^30^ Older age could have a great potential related to both the occurrence of large colorectal lesions and GC. A recent meta‐analysis showed that the risk of GC was 46% and 14% higher in individuals with diabetes mellitus than in those without diabetes mellitus.^31^ In the current study, older age and diabetes mellitus were not significant risk factors of GC probably due to limited numbers.

*H. pylori* infection has been reported as a definite risk factor for the development of GC. However, the incidence of GC after *H. pylori* eradication in Japan was still relatively high after treatment for GC, varying from 4% to 13% after a median follow‐up survey of 4.5 years.^10^, ^11^, ^12^, ^32^, ^33^ Another study reported that the risk increased 9.3 times if the atrophy was severe.^33^ Additionally, there are also several unique reports suggesting that *H. pylori* infection increases the risk of colorectal adenoma.^34^, ^35^, ^36^ Moreover, a systemic review reported a positive association (odds ratio: 1.70, 95% CI: 1.64–1.76) between *H. pylori* infection and CRC.^37^ The rationale of this was suspected that chronic gastritis due to *H. pylori* infection can increase gastrin production and induce systemic inflammation by increasing the production and activity of inflammatory markers such as cyclooxygenase‐2.^38^ Thus, these changes may promote colorectal neoplasia. Recent research has elucidated the detailed mechanisms by which *H. pylori* infection promotes CRC.^39^ *H. pylori* infection induces immune alterations, characterized by a reduction in regulatory T cells and pro‐inflammatory T cells. It also activates pro‐carcinogenic STAT3 signaling and leads to the loss of goblet cells. These factors are thought to contribute to tumor development. Large colorectal lesions have been identified as a risk factor for GC in the current and previous studies, which may be associated with *H. pylori* infection.^15^ Further investigation is warranted to confirm the relationship between large colorectal lesions and the development of GC.

Compared with the GC cases before colorectal ESD, the GC cases after colorectal ESD were significantly smaller and the tumor depth was less invasive. Because EGD was performed for screening after colorectal ESD in our study and most cases were asymptomatic. In Japan, radiography or EGD for screening as GC is recommended for patients ≥50 years old every 2 years. However, the participant rate was reported 48.4% in 2022.^40^ Thus, we suggest the resection of colorectal lesions ≥20 mm should be another option for people to receive EGD for screening.

Regarding limitations, this was a single‐center, retrospective study with a small number of cases in Japan. About one‐third of the patients refused EGD after colorectal ESD. Data about patients and lesion characteristics were missing in some patients. *H. pylori* infection and atrophic gastritis were examined in limited cases. Four cases with GC after colorectal ESD received EGD in other institutions and medical records only about GC were achieved. Thus, the existence of atrophic gastritis could not be examined. The number of *H. pylori* eradication was not examined. The distinction between simultaneous and metachronous GC could not be clarified in the current study. Because we did not perform EGD before colorectal ESD in cases with GC after colorectal ESD. We utilized national cancer registries, but information on the presence of large tumors was not included in the registry data. The comparison of the incidence of GC with that of a colorectal tumor‐free population who underwent EGD at our institution, using matching based on age, sex, and *H. pylori* infection status, should be performed in our future study. However, the prevalence of large colorectal tumors (≥20 mm) was notably low in our prior study.^15^ Specifically, the treatment rate for colorectal tumors ≥20 mm was only 4.9% among all colorectal endoscopic resections (3065/62,392), suggesting that such lesions are quite rare in the general population. Therefore, we believe that this low prevalence did not significantly affect our analysis in comparison to the national cancer registry data. The *H. pylori* eradication has been widely performed since 2013 as approved by the health insurance system and this led to a decrease in the incidence of GC in the Japanese population.^2^

## CONCLUSION

The GC rate after resecting colorectal lesions ≥20 mm with ESD was 5.96%, suggesting the necessity of EGD to find GC in the early stage regardless of symptoms.

## CONFLICT OF INTEREST STATEMENT

Naohisa Yoshida and Osamu Dohi received a research grant from Fujifilm. Naohisa Yoshida received payment for lectures from Fujifilm. The other authors declare no conflict of interest for this article.

## ETHICS STATEMENT

This study was conducted following the World Medical Association Declaration of Helsinki and was approved by the institutional review board and ethics committees of Kyoto Prefectural University of Medicine (ERB‐C‐1944). This study was retrospective, and the opt‐out approach was adopted for informed consent, using their website or a notice in the posting area. Registry and the Registration No. of the study/trial: N/A. Animal Studies: N/A.

## Supporting information




**Supporting Figure A case of gastric cancer (T1) after colorectal endoscopic submucosal dissection**. (a) 63‐year‐old man, 0‐IIa, 30mm, rectum; (b) ESD, en bloc resection; (c) Histopathology: well‐differentiated adenocarcinoma, Tis, horizontal and vertical margin (‐); (d) The patient initially refused EGD and received EGD 5 years after colorectal ESD. The lesion was 0‐IIa, 12mm, middle body (black arrow). Atrophic gastritis: positive; (e) ESD, en bloc resection; (f) Histopathology: well‐differentiated adenocarcinoma, T1 (450 µm), lymphovascular invasion (‐), and horizontal and vertical margin (‐).

## References

[1] World Health Organization, International Agency for Research on Cancer . Factsheets [Internet]. 2020. [Cited 2024 April 29]. Available from: https://gco.iarc.fr/today/online‐analysis‐table

[2] National Cancer Center Japan . Cancer Incidence National Cancer Registry in Japan *[Internet]*, Tokyo: Ministry of Health, Labour and Welfare, 2024. Available from: https://ganjoho.jp/reg_stat/statistics/data/dl/en.html

[3] Tanaka S , Kashida H , Saito Y *et al*. Japan Gastroenterological Endoscopy Society guidelines for colorectal endoscopic submucosal dissection/endoscopic mucosal resection. Dig Endosc 2020; 32: 219–239. 31566804 10.1111/den.13545

[4] Nakajima T , Saito Y , Tanaka S *et al*. Current status of endoscopic resection strategy for large, early colorectal neoplasia in Japan. Surg Endosc 2013; 27: 3262–3270. 23508817 10.1007/s00464-013-2903-x

[5] Benson AB , Venook AP , AI‐Hawary MM *et al*. Colon Cancer, Version 2.2021, NCCN Clinical Practice Guidelines in Oncology. J Natl Compr Canc Netw 2021; 19: 329–359. 33724754 10.6004/jnccn.2021.0012

[6] Yoshino T , Argilés G , Oki E *et al*. Pan‐Asian adapted ESMO Clinical Practice Guidelines for the diagnosis treatment and follow‐up of patients with localized colon cancer. Ann Oncol 2021; 32: 1496–1510. 34411693 10.1016/j.annonc.2021.08.1752

[7] Hassan C , Wysocki PT , Fuccio L *et al*. Endoscopic surveillance after surgical or endoscopic resection for colorectal cancer: European Society of Gastrointestinal Endoscopy (ESGE) and European Society of Digestive Oncology (ESDO) Guideline. Endoscopy 2019; 51: 266–277. 30722071 10.1055/a-0831-2522

[8] Hashiguchi Y , Muro K , Saito Y *et al*. Japanese Society for Cancer of the Colon and Rectum (JSCCR) guidelines 2019 for the treatment of colorectal cancer. Int J Clin Oncol 2020; 25: 1–42. 31203527 10.1007/s10147-019-01485-z PMC6946738

[9] Kawamura T , Wada H , Sakiyama N *et al*. Examination time as a quality indicator of screening upper gastrointestinal endoscopy for asymptomatic examinees. Dig Endosc 2017; 29: 569–575. 28066945 10.1111/den.12804

[10] Fukase K , Kato M , Kikuchi S *et al*. Effect of eradication of *Helicobacter pylori* on incidence of metachronous gastric carcinoma after endoscopic resection of early gastric cancer: An open‐label, randomized controlled trial. Lancet 2008; 372: 392–397. 18675689 10.1016/S0140-6736(08)61159-9

[11] Kamada T , Hata J , Sugiu K *et al*. Clinical features of gastric cancer discovered after successful eradication of *Helicobacter pylori*: Results from a 9‐year prospective follow‐up study in Japan. Aliment Pharmacol Ther 2005; 21: 1121–1126. 15854174 10.1111/j.1365-2036.2005.02459.x

[12] Mori G , Nakajima T , Asada K *et al*. Incidence of and risk factors for metachronous gastric cancer after endoscopic resection and successful *Helicobacter pylori* eradication: Results of a large‐scale, multicenter cohort study in Japan. Gastric Cancer 2016; 19: 911–918. 26420267 10.1007/s10120-015-0544-6

[13] World Cancer Research Fund/American Institute for Cancer Research . Continuous Update Project Report: Diet, Nutrition, Physical Activity and Colorectal Cancer *[Internet]*, 2017, [Cited 2022 Dec 12]. Available from: https://www.wcrf.org/sites/default/files/Colorectal‐cancer‐report.pdf

[14] Matsuo K , Mizoue T , Tanaka K *et al*. Association between body mass index and the colorectal cancer risk in Japan: Pooled analysis of population‐based cohort studies in Japan. Ann Oncol 2012; 23: 479–490. 21597097 10.1093/annonc/mdr143

[15] Yoshida N , Maeda‐Minami A , Ishikawa H *et al*. Analysis of the development of gastric cancer after resecting colorectal lesions using large‐scale health insurance claims data. J Gastroenterol 2023; 58: 1105–1113. 37646980 10.1007/s00535-023-02035-1

[16] Satouchi M , Negoro S , Funada Y *et al*. Predictive factors associated with prolonged survival in patients with advanced non‐small‐cell lung cancer (NSCLC) treated with gefitinib. Br J Cancer 2007; 96: 1191–1196. 17387341 10.1038/sj.bjc.6603710 PMC2360147

[17] World Health Organization . International Guide for Monitoring Alcohol Consumption and Related Harm. Geneva: WHO; 2000.

[18] Masaoka H , Ito H , Gallus S *et al*. Combination of ALDH2 and ADH1B polymorphisms is associated with smoking initiation: A large‐scale cross‐sectional study in a Japanese population. Drug Alcohol Depend 2017; 173: 85–91. 28212515 10.1016/j.drugalcdep.2016.12.015

[19] Lambert R , Lightdale CJ . The Paris endoscopic classification of superficial neoplastic lesions: Esophagus, stomach, and colon. Gastrointest Endosc 2003; 58: S3–43. 14652541 10.1016/s0016-5107(03)02159-x

[20] Kimura K , Takemoto T . An endoscopic recognition of the atrophic border and its significance in chronic gastritis. Endoscopy 1969; 1: 87–97.

[21] WHO Classification of Tumors Editorial Board . WHO Classification of Tumors of Digestive System Tumors, 5th edn, Lyon: International Agency for Research on Cancer, 2019.

[22] Japanese Society for Cancer of the Colon and Rectum . Japanese classification of colorectal, appendiceal, and anal carcinoma: The 3rd English edition [secondary publication]. J Anus Rectum Colon 2019; 3: 175–195. 31768468 10.23922/jarc.2019-018 PMC6845287

[23] Japanese Gastric Cancer Association . Japanese classification of gastric carcinoma: 3rd English edition. Gastric Cancer 2011; 14: 101–112. 21573743 10.1007/s10120-011-0041-5

[24] Noura S , Ohue M , Seki Y *et al*. Second primary cancer in patients with colorectal cancer after a curative resection. Dig Surg 2009; 26: 400–405. 19923828 10.1159/000229991

[25] Lim SB , Jeong SY , Choi HS *et al*. Synchronous gastric cancer in primary sporadic colorectal cancer patients in Korea. Int J Colorectal Dis 2008; 23: 61–65. 17724601 10.1007/s00384-007-0366-z

[26] van den Bussche H , Schön G , Kolonko T *et al*. Patterns of ambulatory medical care utilization in elderly patients with special reference to chronic diseases and multimorbidity—Results from a claims data based observational study in Germany. BMC Geriatr 2011; 11: 54. 21914191 10.1186/1471-2318-11-54 PMC3180370

[27] Tak DH , Moon HS , Kang SH *et al*. Prevalence and risk Factors of gastric adenoma and gastric cancer in colorectal cancer patients. Gastroenteol Res Pract 2016; 2016: 2469521. 10.1155/2016/2469521 PMC5220511 28105047

[28] Yoshida N , Naito Y , Siah KT *et al*. High incidence of metachronous advanced adenoma and cancer after endoscopic resection of colon polyps ≥20 mm in size. Dig Endosc 2016; 28: 194–202. 26422700 10.1111/den.12551

[29] Smith AM , Watson SA . Review article: Gastrin and colorectal cancer. Aliment Pharmacol Ther 2000; 14: 1231–1247. 11012467 10.1046/j.1365-2036.2000.00842.x

[30] Thrift AP , Nguyen TH . Gastric cancer epidemiology. Gastrointest Endosc Clin N Am 2021; 31: 425–439. 34053631 10.1016/j.giec.2021.03.001

[31] Guo J , Liu C , Pan J *et al*. Relationship between diabetes and risk of gastric cancer: A systematic review and meta‐analysis of cohort studies. Diabetes Res Clin Pract 2022; 187: 109866. 35398143 10.1016/j.diabres.2022.109866

[32] Ami R , Hatta W , Iijima K *et al*. Factors associated with metachronous gastric cancer development after endoscopic submucosal dissection for early gastric cancer. J Clin Gastroenterol 2017; 51: 494–499. 27505404 10.1097/MCG.0000000000000620

[33] Shichijo S , Hirata Y , Niikura R *et al*. Association between gastric cancer and the Kyoto classification of gastritis. J Gastroenterol Hepatol 2017; 32: 1581–1586. 28217843 10.1111/jgh.13764

[34] Rokkas T , Sechopoulos P , Pistiolas D *et al*. The relationship of *Helicobacter pylori* infection and colon neoplasia, on the basis of meta‐analysis. Eur J Gastroenterol Hepatol 2013; 25: 1286–1294. 23820245 10.1097/MEG.0b013e328363d3cd

[35] Nam JH , Hong CW , Kim BC *et al*. *Helicobacter pylori* infection is an independent risk factor for colonic adenomatous neoplasms. Cancer Causes Control 2017; 28: 107–115. 28025763 10.1007/s10552-016-0839-x

[36] Sonnenberg A , Genta RM . *Helicobacter pylori* is a risk factor for colonic neoplasms. Am J Gastroenterol 2013; 108: 208–215. 23208272 10.1038/ajg.2012.407

[37] Zuo Y , Jing Z , Bie M *et al*. Association between Helicobacter pylori infection and the risk of colorectal cancer: A systematic review and meta‐analysis. Medicine 2020; 99: e21832. 32925719 10.1097/MD.0000000000021832 PMC7489651

[38] Selgrad M , Bornschein J , Kandulski A *et al*. Helicobacter pylori but not gastrin is associated with the development of colonic neoplasms. Int J Cancer 2014; 135: 1127–1131. 24496701 10.1002/ijc.28758

[39] Ralser A , Dietl A , Jarosch S *et al*. Helicobacter pylori promotes colorectal carcinogenesis by deregulating intestinal immunity and inducing a mucus‐degrading microbiota signature. Gut 2023; 72: 1258–1270. 37015754 10.1136/gutjnl-2022-328075

[40] Ministry of Health, Labour and Welfare . Regional health and health promotion project report [Internet]. 2020 [cited 2023 February 14]. Available from: https://www.mhlw.go.jp/stf/seisakunitsuite/bunya/0000059490.html

