# Supplementary figures and images for: Prevalence of gastric cancer following colorectal endoscopic submucosal dissection for lesions more than 20 mm: A retrospective analysis
Source: DEN Open. 2024 Dec 18;5(1):e70042. doi: 10.1002/deo2.70042 (PMC11653160; doi:10.1002/deo2.70042)

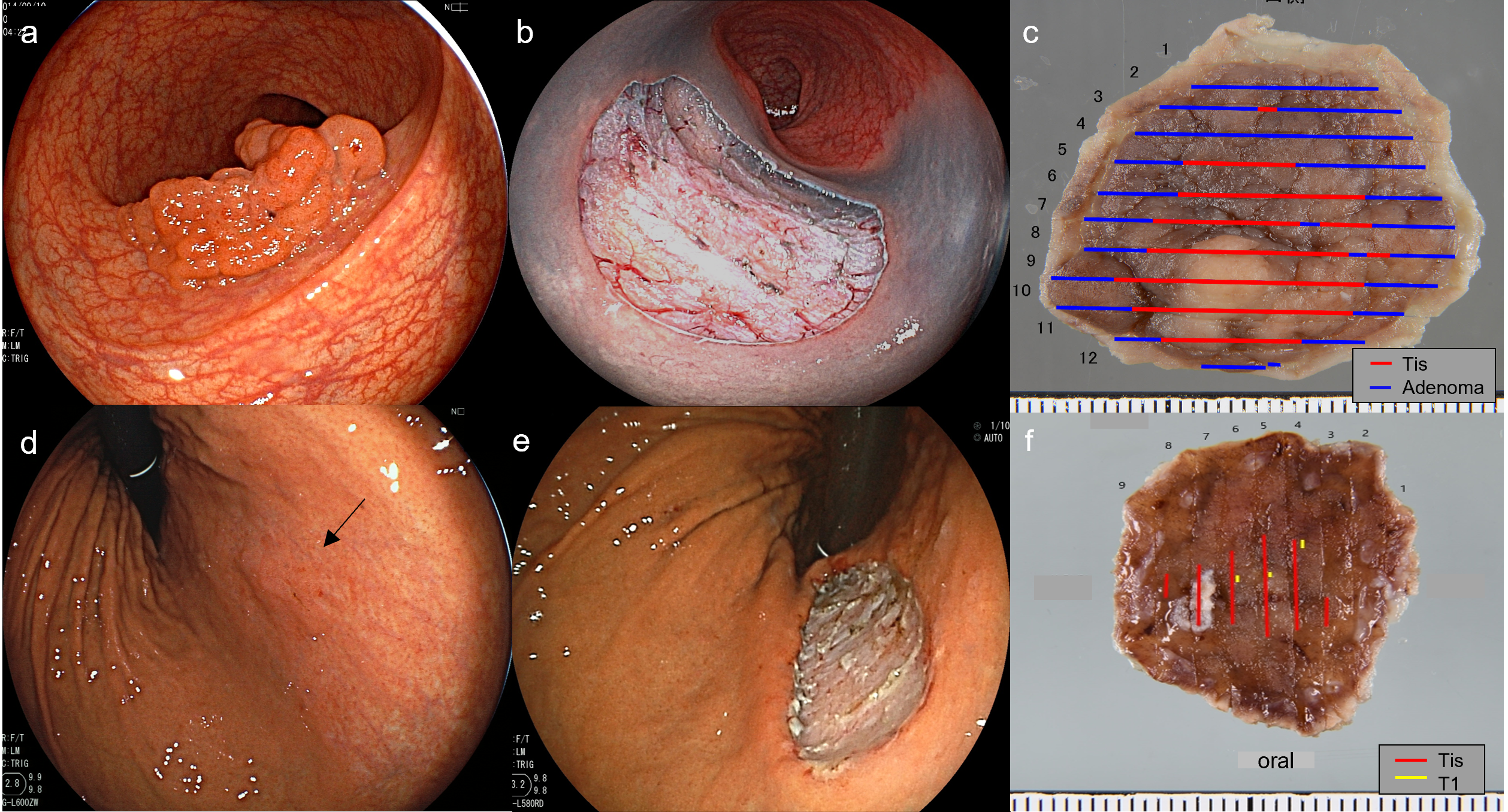

Supplement: Supplementary file 1 — Supporting Figure A case of gastric cancer (T1) after colorectal endoscopic submucosal dissection. (a) 63‐year‐old man, 0‐IIa, 30mm, rectum; (b) ESD, en bloc resection; (c) Histopathology: well‐differentiated adenocarcinoma, Tis, horizontal and vertical margin (‐); (d) The patient initially refused EGD and received EGD 5 years after colorectal ESD. The lesion was 0‐IIa, 12mm, middle body (black arrow). Atrophic gastritis: positive; (e) ESD, en bloc resection; (f) Histopathology: well‐differentiated adenocarcinoma, T1 (450 µm), lymphovascular invasion (‐), and horizontal and vertical margin (‐). [file DEO2-5-e70042-s001.tif]
